# Bioinspired Metastable Transient Peeling for Repeatable Rapid Release

**DOI:** 10.1002/advs.77766

**Published:** 2026-09-16

**Authors:** Lvzhou Li, Qingyue Li, Yao Hu, Xu Dong, Xiangli Wen, Kai Zhu, Yaoyao Jiang, Yu Tian, Jianning Ding

**Affiliations:** ^1^ Institute of Technology for Carbon Neutralization Yangzhou University Yangzhou People's Republic of China; ^2^ Key Laboratory of Synthetic and Biological Colloids Ministry of Education School of Chemical and Material Engineering Jiangnan University Wuxi People's Republic of China; ^3^ State Key Laboratory of Tribology in Advanced Equipment Tsinghua University Beijing People's Republic of China

**Keywords:** bioinspired adhesion, damage‐free wafer handling, metastable transient peeling, reversible adhesion, viscoelastic gripper

## Abstract

Reversible adhesives must hold securely yet release quickly, but conventional designs regulate adhesion only in the strength domain, leaving holding and release in direct conflict. This trade‐off limits damage‐free handling of ultrathin, brittle materials such as silicon wafers. Inspired by the explosive seed ejection of the squirting cucumber (*Ecballium elaterium*), we introduce metastable transient peeling (MTP), which regulates when stored interfacial energy is released. A preload establishes a metastable contact with a tunable energy barrier, and a minute perturbation then triggers self‐accelerated, edge‐synchronous peeling; a four‐parameter mechanics framework links material and operating variables to performance. A viscoelastic hemispherical gripper releases within 52 ms, retains over 85% of its release performance after 300 000 cycles, and performs 10 000 damage‐free pick‐up, transfer, and release operations on 45‐µm‐thick silicon wafers while remaining operative at 10 Pa. MTP establishes temporal‐domain control as a route to fast, clean, repeatable release.

## Graphical abstract

Inspired by the explosive seed ejection of the squirting cucumber, this work introduces metastable transient peeling that temporally regulates stored interfacial energy release via a tunable energy barrier. A viscoelastic hemispherical gripper achieves 52 ms ultrafast, residue‐free release with 300 000‐cycle durability, enabling damage‐free handling of ultrathin brittle silicon wafers for high‐throughput manufacturing.

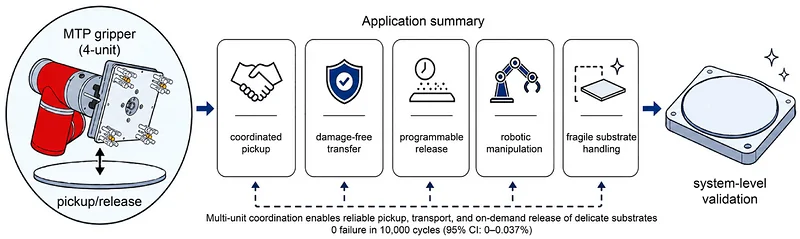

## 1 Introduction

Reversible adhesion underpins technologies as diverse as soft robotic grippers, transfer printing of flexible electronics, and the high‐throughput handling of semiconductor wafers. Bioinspired strategies have advanced this capability rapidly, ranging from dry adhesion based on gecko setae to wet adhesion inspired by octopus suckers [1, 2, 3, 4, 5, 6, 7, 8, 9, 10]. By replicating characteristic microstructures and material gradients, these systems grip without chemical bonding agents [11, 12, 13, 14, 15], which avoids the surface contamination and mechanical damage typical of conventional handling.

Most existing designs operate in a single regime. They regulate adhesion in the strength domain, switching between gripping and release by changing how strongly the interface resists separation, whether through the material modulus [16, 17, 18, 19], the deformation of surface microstructures [20, 21, 22, 23], or applied external fields [24, 25, 26, 27]. Even recent metamaterial adhesives that program adhesion through reverse crack propagation [28] and snap‐through metastructures for micro‐object manipulation [29] remain governed by strength‐domain mechanics: they tune how strongly or how directionally an interface resists separation, not when its stored energy is released. Because these mechanisms only adjust how strongly an interface resists separation, they face an intrinsic trade‐off: strengthening adhesion makes detachment harder, whereas prioritizing fast release sacrifices holding stability or load capacity. The result is a missing temporal axis. Strength‐domain control answers how strongly an interface resists separation; it offers no handle on when stored interfacial energy is discharged. In many representative systems, release times remain on the order of hundreds of milliseconds or longer, limiting their use in high‐throughput handling [30, 31, 32, 33].

Nature takes a different route: rather than balancing holding against release, it changes the regulated variable, controlling the timing of energy release instead of the strength of the bond. The seed‐ejection mechanism of the squirting cucumber illustrates this [34]. Elastic energy stored in the pressurized fruit wall is held at a critical state by the constrained stalk and discharged the instant the constraint is removed. Recent biomechanical work has shown that turgor‐driven pressurization combined with geometric constraint produces a precisely tuned mechanical instability [35, 36]. This sequence of energy storage, critical state, and triggered release enables ultrafast ejection, but it is inherently single‐use: the plant cannot choose when to fire, and the mechanism cannot be reset.

We translate this energy sequence into an engineered, repeatable adhesive interface, termed metastable transient peeling (MTP). Unlike conventional rate‐dependent viscoelastic detachment, preload‐modulated peeling, and snap‐off instability—all of which operate within the strength domain where the external load continuously drives crack propagation, and the peel force scales monotonically with the trigger rate as a direct consequence of the rate‐dependent material modulus [37, 38]—MTP decouples energy storage from energy release along the time axis. Retaining the storage, trigger, and release logic of the squirting cucumber while adding on‐demand triggering and cyclability, MTP replaces the plant's passive critical equilibrium with an engineered mechanical metastable state possessing a tunable energy barrier. The cycle comprises four phases: preloading fully injects elastic energy into the interface; unloading locks this energy behind the tunable barrier; a minute triggering perturbation supplies only a small fraction of the total peeling energy to cross the barrier; and self‐accelerated peeling then proceeds autonomously without further external energy input, accompanied by an autonomous reset (Table S1). Across this cycle, energy is discharged only upon the application of a minute perturbation, affording direct control over the timing of release rather than the strength of the bond.

We realize this principle in a viscoelastic hemispherical MTP gripper. The gripper releases within 52 ms, retains more than 85% of its release performance after 300 000 cycles, completes 10 000 damage‐free pick‐up, transfer, and release operations on 45‐µm‐thick silicon wafers, and operates stably under low pressure (*F*_c_ fluctuation < 6% at 10 Pa) and across 10°C–50°C (*F*_c_ fluctuation < 10%). A mechanics‐guided framework connects these metrics to material and geometric design, which establishes temporal‐domain regulation as a general strategy for reversible adhesion.

## 2 Results

### 2.1 Bioinspired Principle and Energy Evolution of MTP

**FIGURE 1:**
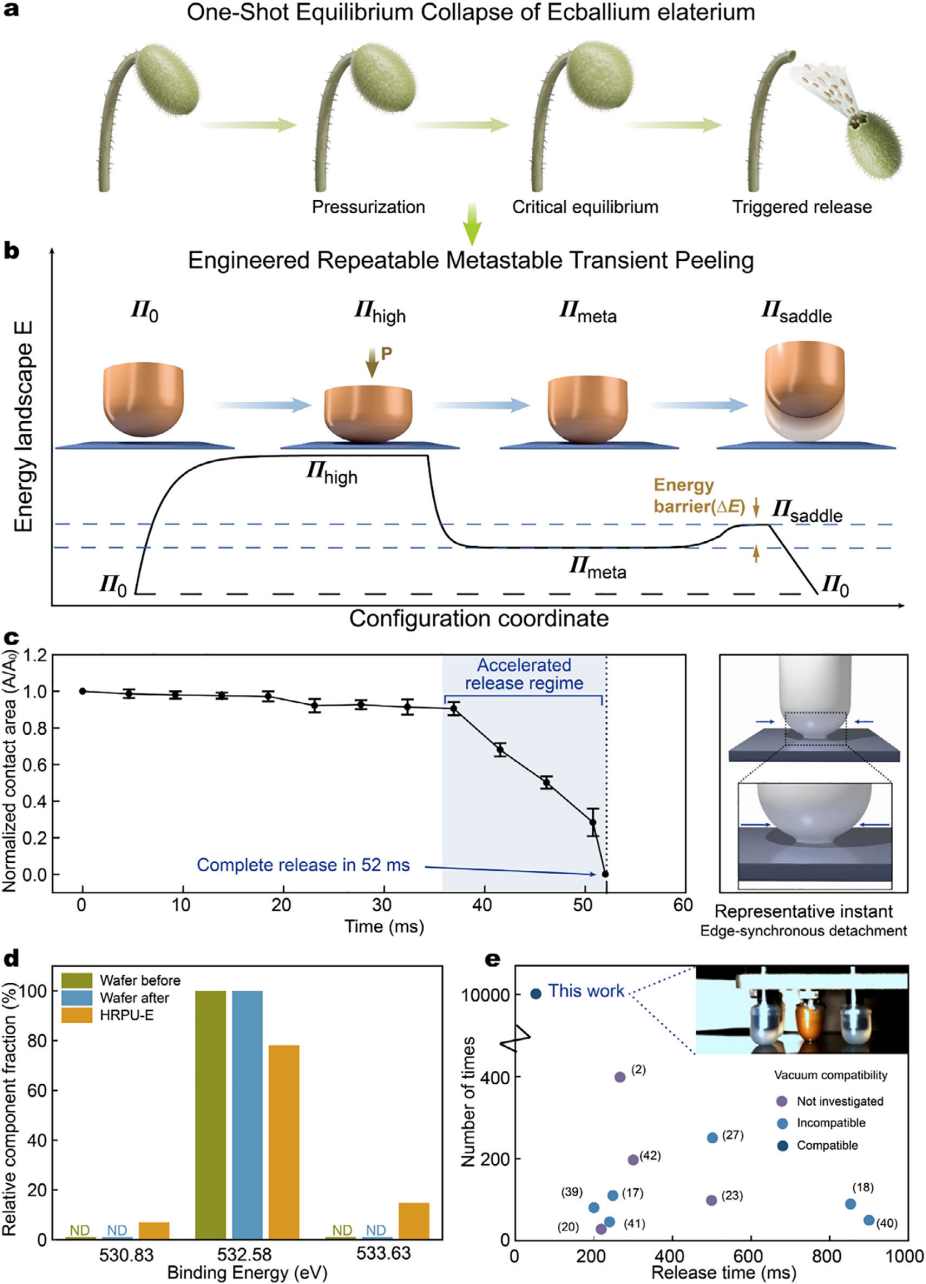
Metastable transient peeling (MTP) concept and first evidence of fast, clean, and repeatable release. (a) Biological inspiration. The squirting cucumber (*Ecballium elaterium*) achieves one‐shot rapid ejection through a sequence of pressurization, critical equilibrium, and triggered release. (b) Engineering translation. Energy pathway of the MTP strategy, showing the energy barrier (Δ*E*), metastable state (*Π*_meta_), and saddle point (*Π*_saddle_). The pathway is schematic and does not represent a calculated potential‐energy surface. (c) Release dynamics. A minute trigger perturbation converts into full‐perimeter synchronous, self‐accelerated peeling and reaches complete detachment within 52 ms. Left, normalized contact area (A/A_0_) over time. Right, schematic of the metastable contact geometry under compressive stress. (d) Interfacial cleanliness. XPS O 1s spectra of the silicon wafer surface before and after MTP release show no detectable hydroxyl‐functionalized rigid polyurethane elastomer (HRPU‐E) characteristic peaks (O─H at 533.6 eV, O═C at 530.8 eV), which supports chemically clean release with no measurable residue transfer. (e) Performance positioning. Benchmarking of MTP against existing adhesive‐release technologies on release time, cycle life, and vacuum compatibility. Data for existing technologies are from refs [2, 17, 18, 20, 23, 27, 39, 40, 41, 42] comparison metrics and test conditions are detailed in Table S2.

To turn this one‐shot biological release into a repeatable interface, we first had to identify the mechanical states that let stored energy be held and then discharged on demand. The cucumber fixes this with a one‐shot collapse of mechanical equilibrium (Figure 1a), which is efficient but passive, because its timing cannot be controlled and the system cannot be re‐established.

We sought to recreate this behavior in an engineered, repeatable adhesive interface, in which a preload‐established metastable contact holds temporarily and then collapses through triggered transient peeling. The interface operates through a four‐stage energy‐evolution sequence (Figure 1b). In the first stage, contact and energy injection, a preload deforms the viscoelastic hemisphere elastically into conformal contact, leaving it in a high‐energy state maintained by the external force. In the second stage, metastable‐state construction, during preloading, the viscoelastic hemisphere is compressed and stores elastic strain energy. Upon removal of the preload, elastic recovery tends to reduce contact area, while interfacial adhesion resists separation. This balance creates a local minimum in the system potential energy, defined as the metastable state *Π*_meta_. A saddle point *Π*_saddle_ exists between the metastable state and the fully detached state, and the energy difference Δ*E* = *Π*_saddle_ − *Π*_meta_ constitutes the energy barrier that prevents spontaneous detachment. Notably, this energy barrier arises from the competition between elastic recovery and interfacial adhesion. In the third stage, barrier crossing and triggered release, a minute perturbation drives the interface across the effective energy barrier into transient peeling, releasing the stored energy. In the fourth stage, the hemisphere relaxes to its ground state (*Π* = 0), completing the cycle. This sequence provides the physical basis for temporal regulation of adhesion and detachment.

Built on this mechanism, the viscoelastic hemispherical MTP gripper couples material viscoelasticity with hemispherical geometry to convert a minute perturbation into a self‐accelerated, full‐perimeter synchronous peel, with a release time of 52 ms (Figure 1c and Video S1). Wafer‐surface XPS after cyclic testing showed no detectable polymer‐residue transfer, supporting chemically clean release (Figure 1d). Benchmarked against existing systems, these metrics place the MTP gripper among the fastest and most durable adhesive‐release technologies reported (Figure 1e and Tables S2) [2, 17, 18, 20, 23, 27, 39, 40, 41, 42].

These observations establish the MTP release behavior but do not yet explain the dynamics behind its rapid peeling. We next examined whether the hemispherical geometry programs the onset and propagation of detachment.

### 2.2 A Mechanics‐Guided Parameter Framework for MTP

What variables govern whether a metastable contact holds, when it releases, and how it degrades? Combining energetic principles with viscoelastic contact mechanics, we identified four quantities that set the conditions for interfacial instability: the energy barrier (Δ*E*) for energy‐storage capacity, the critical triggering force (*F*_c_) for triggering conditions, the holding‐force decay rate (*η*) for temporal stability, and the hysteresis coefficient (*H*) for cyclic dissipation.

Δ*E* is the minimum energy required to escape the metastable state, and it measures the depth of the metastable well. *F*_c_ is the minimum external force needed to trigger interfacial instability. *η* is the rate at which the holding force decays over time, and it reflects the temporal stability of the metastable state. *H* is the ratio of dissipated energy to total input work over a loading–unloading cycle, reflecting loading–unloading irreversibility and cyclic dissipation. Throughout, we distinguish the holding force, the quasi‐static load that the metastable contact can sustain before release, from the critical triggering force *F*_c_, the small external perturbation that initiates detachment; these are independent quantities, and a useful MTP interface combines a high holding force with a low *F*_c_.

For the contact system between a viscoelastic hemisphere and a rigid substrate, dimensional analysis and Hertzian contact theory (Note S1, Tables S3 and S4) yield the scaling relationship:

(1) $$\Delta E\propto \frac{\gamma^{5/3}R^{4/3}}{{\left[E_{eff}\left(\dot{\varepsilon }\right)\right]}^{2/3}}$$

where *R* is the hemisphere radius and $\dot{\varepsilon }$ is the loading rate. *E*_eff​_ ($\dot{\varepsilon }$) is the rate‐dependent effective modulus. Equation (1) indicates that Δ*E* increases with the interfacial energy *γ* and the hemisphere radius *R* but decreases with increasing *E*_eff_ — that is, a softer material stores more elastic energy after unloading, constructing a deeper metastable well. The scaling of Δ*E* with modulus—in which softer materials yield deeper metastable wells under identical preload—highlights an underexplored design space for adhesive interfaces, as conventional strength‐domain designs typically prioritize stiffer materials for higher load capacity.

On the basis of energy balance and dimensional analysis (Note S2 and Tables S5), the scaling relationship for *F*_c_ is:

(2) $$F_{c}\propto \gamma^{1-\kappa }R^{1+\kappa }{\left(E_{eff}\right)}^{\kappa }$$

where *κ* is the metastability index (0 < *κ* < 1), an empirical parameter that requires experimental calibration. It quantifies the deviation from classical JKR behavior: a larger *κ* indicates that more mechanical energy is locked within the metastable state, requiring greater external force to trigger detachment.

Under small‐deformation conditions near the metastable state, the standard linear solid model describes the stress relaxation behavior (Note S3 and Tables S6):

(3) $$\eta =\frac{1}{\tau }\left(1-\frac{E_{\infty }}{E_{0}}\right)$$

where *τ* is the characteristic relaxation time, *E*_0_ is the instantaneous modulus, and *E*_∞_ is the equilibrium modulus. The value of *η* is determined by the material's intrinsic viscoelastic parameters.

*H* comprises two components: viscoelastic dissipation and dissipation from the metastable energy barrier (Note S4 and Tables S7):

(4) $$H=\beta_{E}\cdot \frac{D_{e}}{1+D_{e}}+\kappa_{2}\frac{\gamma^{2/3+\kappa }R^{1/3-\kappa }}{{\left[E_{eff}\right]}^{2/3+\kappa }\delta_{\max}}$$

where the first term represents pure viscoelastic contribution, with *β_E_* = (*E*_0_ − *E*_∞_)/*E*_0_ being the modulus attenuation coefficient and *D*_e_ being the Deborah number. The second term accounts for metastable energy barrier dissipation, with *κ*_2_ determined experimentally. Under quasistatic conditions, metastable dissipation predominates; under high‐speed conditions, viscoelastic dissipation becomes dominant.

This framework provides a design language connecting material properties, geometric parameters, and operating conditions to measurable MTP performance. The scaling relationships capture the observed trends in our experimental data and guide the selection of material formulations and operating parameters.

### 2.3 Metastable Hold and Triggered Synchronous Peeling

**FIGURE 2:**
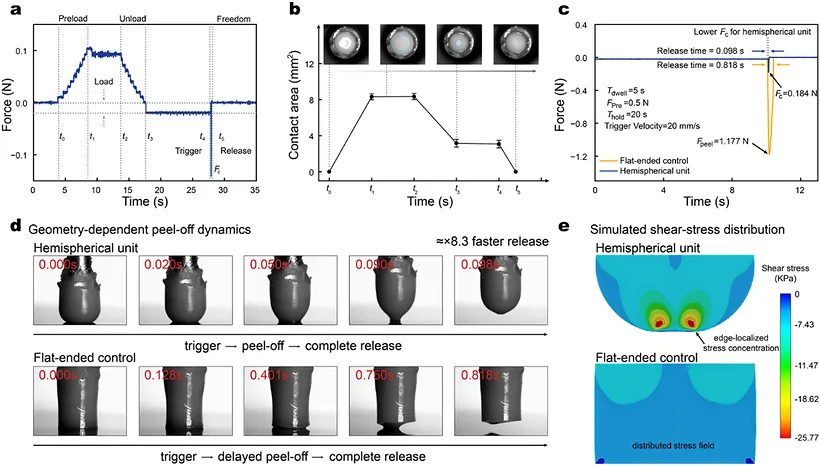
Hemispherical geometry programs edge‐synchronous peeling, which reduces the triggering force and accelerates release. (a) Test procedure for a single MTP adhesive unit: loading for energy storage, unloading for metastable‐state construction, and triggering for release. (b) Contact‐area evolution during the procedure. The hemisphere enters a low‐contact‐area metastable state after unloading, whereas a cylindrical control does not. (c) Quantitative comparison. The hemispherical unit reduces F_c_ to 15.6% of that of the cylindrical control (0.184 N vs. 1.177 N) and shortens the release time to 12.0% (0.098 s vs. 0.818 s). (d) High‐speed image sequence (Video S3). The hemisphere shows full‐perimeter synchronous crack initiation and inward propagation, while the cylinder shows random sequential cracking. (e) Stress distribution from finite‐element analysis (Note S5 and Figure S6). Stress concentrates at the contact perimeter for the hemisphere, consistent with the observed edge‐synchronous detachment, whereas the cylinder shows a uniform, low‐stress distribution. Quantitative data in (c) are mean ± SD (*n* ≥ 5).

To test the framework, we first investigated whether a hemispherical contact actually enters a metastable state on unloading and how that state changes the force and time needed to detach. We synthesized a hydroxyl‐functionalized rigid polyurethane elastomer (HRPU‐E), molded it into hemispheres of 2.5 mm radius, and used solar‐grade silicon wafers as substrates (*S*_a_ = 0.4131 µm; Figure S1). The test protocol and working‐state definitions for a single adhesive unit are shown in Figure 2a.

HRPU‐E was polymerized from polyether polyol and isophorone diisocyanate (IPDI); its surface hydroxyl groups arise from residual moieties of the polyol and require no post‐functionalization (synthetic route, Figure S2; fabrication, Figure S3). Fourier‐transform infrared spectroscopy (Figure S4) showed characteristic carbamate and urea absorptions at 3313.58 and 1701.87 cm^−1^, consistent with successful synthesis. Deconvolution of the high‐resolution O 1s spectrum (Figure S5) resolved three peaks, O═C (530.83 eV), O─C (532.28 eV), and O─H (533.58 eV), consistent with surface hydroxyl and polar groups that may support reversible polar interactions with the native silicon oxide surface [43, 44, 45]. Integration of the O─H component gives a relative O─H fraction of about 14.8%.

The metastable hold was first evident in the contact‐area evolution upon unloading. During loading–unloading cycles (Video S2), the contact area of the hemisphere fell sharply after preload removal (Figure 2b), whereas that of a cylindrical control was nearly unchanged, consistent with the hemisphere, but not the cylinder, entering a low‐contact‐area metastable state.

This metastable hold sharply lowers both the detachment force and the detachment time. Under a preload of 0.5 N with the contact area controlled to be identical, the viscoelastic hemisphere has a critical triggering force of 0.184 N, which is 15.6% of the cylinder's value of 1.177 N, and a release time of 0.098 s, which is 12.0% of the cylinder's 0.818 s (Figure 2c). This comparison removes the confounding effect of nominal contact area and confirms that edge stress localization, not contact area itself, enables fast low‐force detachment. High‐speed imaging (Figure 2d and Video S3) reveals distinct crack behavior. In the hemisphere, cracks initiate simultaneously around the entire perimeter and form a closed loop that propagates rapidly inward, and the contact diameter shrinks at an accelerating rate, consistent with self‐accelerated peeling. The cylinder, by contrast, shows random crack initiation and far slower peeling. Finite‐element analysis shows stress concentrating at the contact perimeter of the hemisphere, whereas the cylinder bears a uniform, low stress (Figure 2e, Figure S6 and Note S5). This stress localization matches the observed edge‐synchronous crack initiation and supports geometry‐induced stress concentration as a mechanical basis for rapid, low‐force detachment.

To further distinguish MTP from conventional rate‐dependent peeling, we conducted constant‐velocity peel tests on matched hemispherical samples and substrates across a 5–200 mm s^−^^1^ loading range as a benchmark. The corresponding force curves, contact area evolution during peeling, and the triggering force *F*_c_ vs. trigger velocity are provided in Figures S7–S9, respectively. In conventional peeling mode, the contact area shows no abrupt contraction, and *F*_c_ scales monotonically with trigger velocity below 50 mm s^−^^1^. In MTP mode, by contrast, the contact area contracts abruptly at a critical trigger instant, while *F*_c_ exhibits only weak velocity dependence below 20 mm s^−^^1^. These results confirm that MTP regulates the timing of energy release rather than the intrinsic interfacial adhesion strength, establishing a clear temporal control strategy fundamentally distinct from conventional rate‐dependent detachment.

### 2.4 A Theory‐Guided Operating Window

**FIGURE 3:**
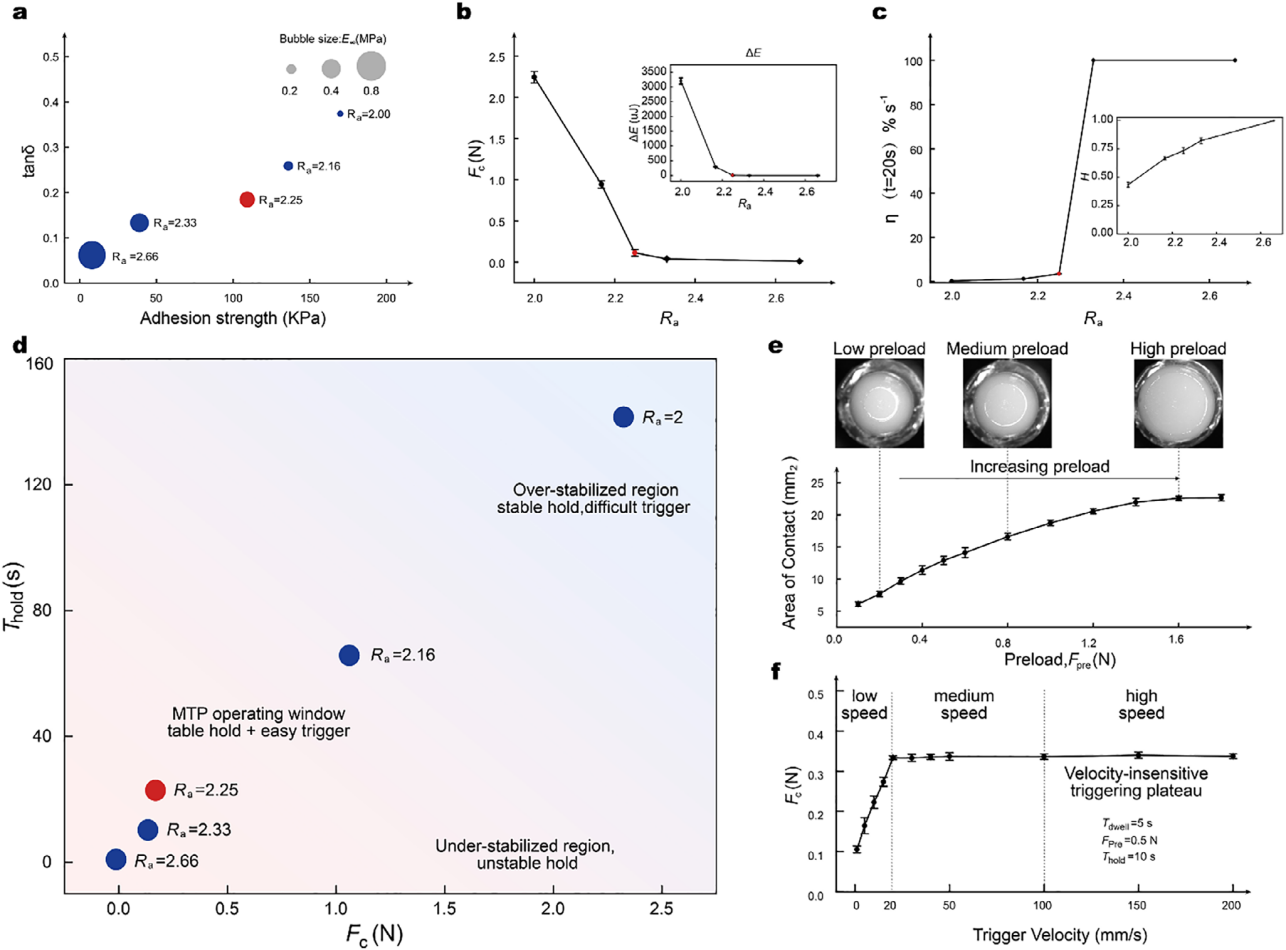
Material and operating parameters define a quantitative MTP operating window that balances holdability and triggerability. (a) Material viscoelastic properties (equilibrium modulus *E*_∞_, adhesion strength *E*_a_, loss factor tan δ) as functions of the molar ratio *R*_a_. (b) Energy barrier ΔE and critical triggering force *F*_c_ vs. *R*_a_. The measured trends are consistent with the scaling relations in Equations (1) and (2). (c) Holding‐force decay rate *η* (*η* is measured at a holding duration of 20 s, with the unit of % per second) and hysteresis coefficient *H* vs. *R*_a_. (d) Operating window. *F*_c_ vs. holding time *T*_hold_ separates the formulations into over‐stabilized (*R*_a_ = 2.00), under‐stabilized (*R*_a_ = 2.66), and operational (*R*_a_ = 2.25) regimes under the tested conditions (preload 0.1 N, loading rate 0.05 mm s^−1^, and triggering velocity 20 mm s^−1^). (e) Effect of preload on the metastable contact area. The corresponding *F*_c_ dependence is shown in Figure S13. (f) Effect of triggering velocity on *F*_c_. Below about 20 mm s^−1^, a relaxation‐assisted regime is observed; above this threshold, *F*_c_ plateaus. Quantitative data are mean ± SD (*n* ≥ 5).

How should an MTP interface be formulated when minimizing *F*_c_ alone would compromise holding stability? We mapped material properties and operating variables to define a functional window that balances hold‐ability and trigger‐ability (Figure 3).

Tuning the molar ratio (*R*_a_) of IPDI to polyol systematically modulates the physical properties of HRPU‐E. We studied its effect on the material properties and on the four MTP parameters (Figure 3a–c, Figures S10–S12, Tables S8 and S9; calculation in Note S6) at a preload of 0.1 N, a loading rate of 0.05 mm s^−1^, and a trigger velocity of 20 mm s^−1^.

At *R*_a_ = 2.00, the adhesion strength (*E*_a_) reaches 167.70 kPa, but the elastic modulus is too low (*E*_∞_ = 0.046 MPa), which gives an excessive energy barrier (Δ*E* = 3.20 × 10^3^ µJ). Although the decay rate is low (*η* = 0.32% s^−1^), the triggering force is impractically large (*F*_c_ = 2.32 N); interfacial filamentation occurs during peeling, and the release is markedly prolonged (Figure S13). The hysteresis coefficient (*H* = 0.44) and loss factor (tan*δ* = 0.38) indicate high viscous dissipation, which is unfavorable for rapid release. At *R*_a_ = 2.66, the modulus is too high (*E*_∞_ = 0.73 MPa), the adhesion strength falls below 1.51 kPa, and Δ*E* becomes negligible (0.002 µJ); despite a vanishing *F*_c_ (0.01 N), *η* approaches 100% s^−1^, indicating rapid loss of measurable holding stability under the tested protocol. At *R*_a_ = 2.25, the balance is optimal: Δ*E* = 6.61 µJ and *F*_c_ = 0.10 N minimize the risk of damaging brittle targets, while *η* = 3.50% s^−1^ reflects good temporal stability. The low loss factor (tan*δ* = 0.18) indicates limited viscous loss and rapid elastic recovery, while the relatively high hysteresis coefficient (*H* = 0.73) is consistent rather than contradictory: *H* quantifies loading–unloading irreversibility, consistent with the level needed to establish the metastable state, whereas tan δ quantifies the viscous loss during fast deformation, so a deep metastable well can coexist with rapid, low‐loss recovery.

To quantify how long the metastable state holds securely, we defined the holding time (*T*_hold_) as the time for the adhesion force to decay to 30% of its initial value, the instability threshold, under quiescent conditions. On a 182 mm × 182 mm, 100‐µm‐thick wafer (Figure S14), *R*_a_ = 2.25 gives *T*_hold_ above 20 s, a sufficient triggering window for the handling protocol used here. By comparison, *R*_a_ = 2.00 gives *T*_hold_ above 140 s, while *R*_a_ = 2.66 gives *T*_hold_ near zero. Plotting *F*_c_ against *T*_hold_ reveals three regimes (Figure 3d): over‐stabilized (*R*_a_ = 2.00), under‐stabilized (*R*_a_ = 2.66), and operational (*R*_a_ = 2.25). This window provides the quantitative basis for designing MTP interfaces.

To directly resolve the metastable state and energy barrier of MTP, we conducted quasi‐static tensile tests at 0.01 mm s^−^^1^ on three HRPU elastomer formulations (*R*_a_ = 2.16, 2.25, and 2.33) at a preload of 0.1 N, reconstructing potential energy–displacement curves via numerical integration with the raw force–time data shown in Figure S15 and the corresponding displacement–time and potential energy–displacement curves in Figures S16 and S17. The metastable state appears as a near‐zero force plateau in the unloading curve after preload removal, marking a local minimum (*Π*_meta_ > 0) in the potential energy profile. Stretching outward from this plateau requires continuous external work, monotonically raising the potential energy to the saddle point (*Π*_saddle_), the maximum along the detachment pathway. Crossing *Π*_saddle_ removes the need for further external work: synchronous cracks nucleate at the hemisphere perimeter, driving self‐accelerated peeling as the potential decays to *Π* = 0. The lower potential of the fully separated state provides the thermodynamic driving force for spontaneous detachment. In Figures S15 and S16, the force drops precipitously to zero immediately after crossing *Π*_saddle_, consistent with rapid inward propagation of a global annular crack. This provides unambiguous kinetic evidence for self‐accelerated peeling in MTP hemispheres. Combined with contact area evolution data from Figure S8, these results form a unified evidence chain linking morphological observation and quasi‐static mechanical quantification, robustly validating the metastable state and energy barrier mechanism underpinning MTP function.

To quantify the passive load‐bearing capacity of the metastable interface, we define the quasi‐static passive holding force *F*_hold_ as the maximum static load sustained under undisturbed conditions. The corresponding test curves extracted from the same quasi‐static measurements are provided in Figure S15. Across a 0.05–0.5 N preload range, *F*_hold_ measures 0.041–0.1475 N (Figure S18), which fully covers the self‐weight range of ultrathin lightweight silicon wafers (4–15 g, corresponding to 0.039–0.147 N). A single *F*_hold_ value cannot fully capture the load‐bearing stability of the metastable interface. We therefore adopt a four‐parameter framework comprising *F*_hold_, *F*_c_, *T*_hold_, and *η* to comprehensively characterize performance. This approach addresses the limitation of relying solely on *F*_hold_ for performance evaluation, and conclusively demonstrates that the MTP gripper delivers stable load‐bearing capacity for ultrathin lightweight silicon wafers.

Preload reshapes the apparent metastable state by changing the initial contact (Figure 3e and Figure S19). A moderate preload reshapes the apparent metastable contact state and improves stability, whereas excessive preload drives *F*_c_ undesirably high (Figure S20). Trigger velocity also affects *F*_c_ (Figure 3f). *F*_c_ rises with trigger velocity and plateaus above 20 mm s^−^^1^. This behavior is viscoelastic in origin: at higher speeds, the effective modulus increases and a larger force is required to overcome Δ*E*. Below 20 mm s^−^^1^, the system enters a relaxation‐assisted regime in which partial stress relaxation lowers the required triggering force. Preload also provides an operational handle on *F*_c_, which can be tuned over a range of 0 to 0.33 N by adjusting the preload force.

On three silicon substrates with surface energies of 60, 66, and 74 mJ m^−^^2^, the *F*_c_ increases monotonically with substrate surface energy (Figure S21).

At a preload of 0.1 N and a trigger velocity of 20 mm s^−1^, the release time falls to 52 ms (Figure 1c and Video S1). The 0.098‐s value reported above reflects the geometry‐comparison condition, whereas the 52‐ms release was achieved under the optimized preload and triggering‐velocity condition. These results support the view that the hemispherical geometry, which provides stress concentration, and the material viscoelasticity, which provides the energy barrier, together establish the mechanical metastability required for rapid, low‐force release.

### 2.5 Interfacial Integrity, Durability, and Environmental Compatibility

Practical robotic handling also requires a clean interface and operational robustness, so we evaluated the optimized MTP unit under repeated cycling, environmental stress, fouling, and pressure variation.

#### 2.5.1 Chemical Cleanliness

**FIGURE 4:**
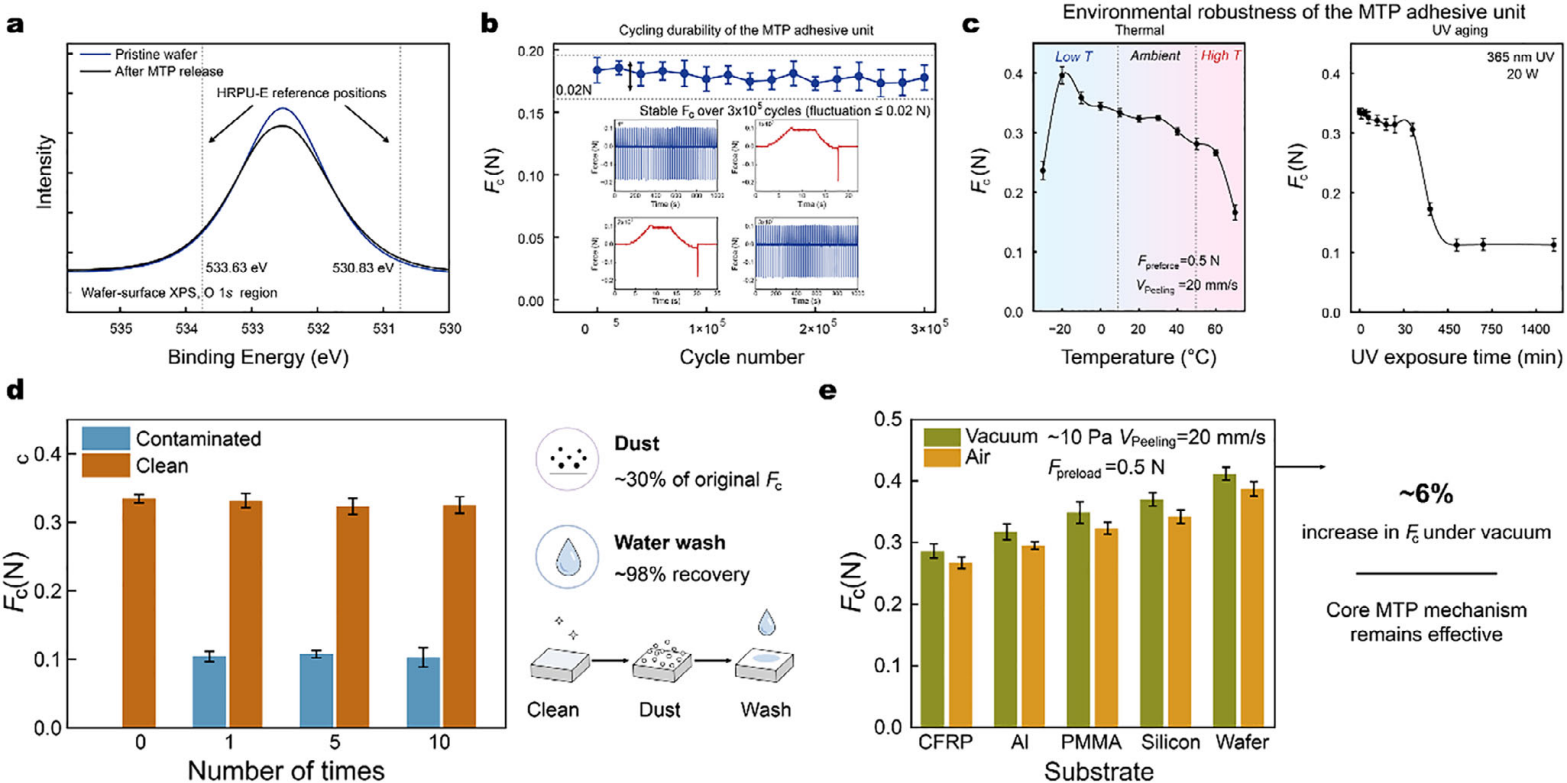
Robustness of the optimized MTP interface under practical perturbations. (a) Interfacial cleanliness. XPS O 1s spectra of the silicon wafer surface before and after MTP release show no detectable HRPU‐E characteristic peaks (O─H at 533.6 eV, O═C at 530.8 eV), which supports chemically clean release with no measurable residue transfer. (b) Cycling durability. *F*_c_ degrades by less than 15% over 300 000 adhesion–detachment cycles (class‐10 000 cleanroom, 22°C, 20% RH). (c) Temperature adaptability. *F*_c_ fluctuates by less than 10% from 10°C to 50°C. UV stability is maintained for 30 min (365 nm, 20 W m^−2^); longer exposure causes progressive degradation, with *F*_c_ reduced by about 70% at 450 min, and prolonged irradiation altered the surface O 1s spectrum (Figure S19). (d) Fouling resistance and cleanability. Dust contamination temporarily reduces F_c_ to 30% of its original value, and water washing restores 98% of the original performance. (e) Low‐pressure compatibility. F_c_ increases by only 6% at 10 Pa compared with ambient pressure, consistent with the MTP release response remaining operative under vacuum conditions. Quantitative data are mean ± SD (*n* ≥ 5).

XPS of the adhesive hemisphere before and after 100 cycles (Figure S22) showed no change in the C 1s shift peaks, which confirms surface chemical stability. Wafer‐surface XPS before and after release gave nearly overlapping O 1s spectra with no peaks at the HRPU‐E reference positions (O─H, 533.6 eV; O═C, 530.8 eV), which indicates chemically clean release with no measurable residue transfer (Figure 4a).

#### 2.5.2 Surface Integrity

Multiscale characterization by white‐light interferometry, scanning electron microscopy (SEM), and photoluminescence (PL) spectroscopy before and after 100 cycles showed the adhesive hemisphere intact and the wafer free of microscratches or microcracks within the inspected contact regions (Figures S23–S25). Because wafers typically undergo only one or a few pick‐up cycles in practice, the 100‐cycle test supports repeated contact without detectable surface damage within the inspected regions.

#### 2.5.3 Cycling Durability

After 300 000 adhesion–detachment cycles (3 independent HRPU‐E hemisphere samples, each tested against an independent silicon wafer) in a class‐10 000 cleanroom (22°C, 20% RH), *F*_c_ (the mean of the three samples) degraded by less than 15% (Figure 4b and Video S4), and the dynamic release was monitored by high‐speed imaging (Video S5). After 50 days of storage, *F*_c_ degraded by less than 5% (Figure S26).

#### 2.5.4 Environmental Robustness

The MTP interface remained stable across diverse challenges (Figure 4c–e). Temperature variation from 10°C to 50°C induced less than 10% *F*_c_ fluctuation, and 30 min of UV exposure left *F*_c_ unchanged, with longer exposure causing gradual degradation (Figure 4c); prolonged irradiation also altered the O 1s spectrum (Figure S27). Dust contamination was reversed by water washing, with 98% recovery (Figure 4d, Figures S28, S29, and Video S6). Low‐pressure operation at 10 Pa increased *F*_c_ by only 6% (Figure 4e), which indicates potential compatibility with vacuum‐based semiconductor processes (Figure S30).

### 2.6 System Integration and Application Validation

**FIGURE 5:**
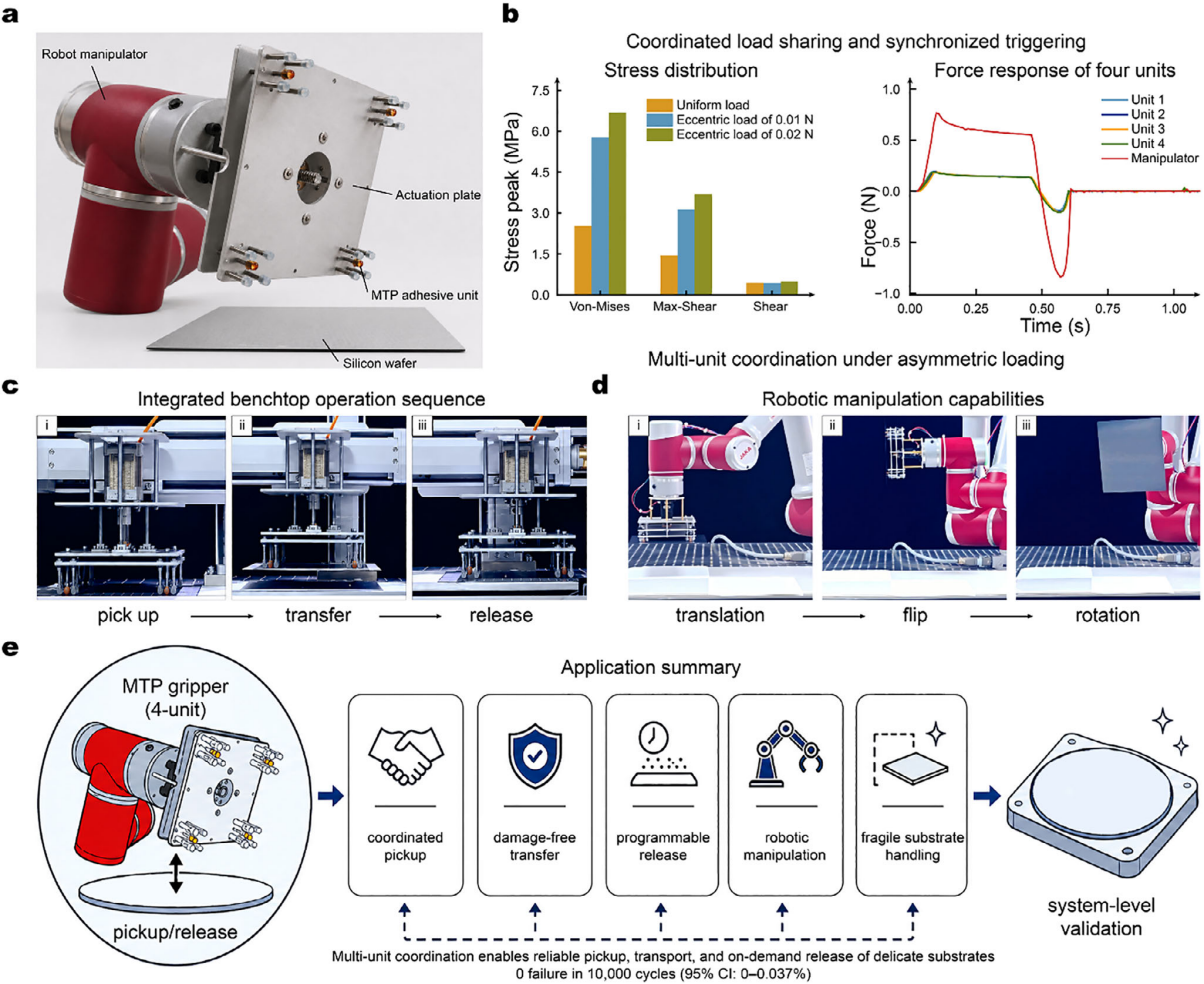
System integration from a single adhesive unit to a four‐unit robotic gripper for fragile‐substrate handling. (a) Mechanical design and layout of the four‐unit MTP gripper, showing the concentric configuration of adhesive and non‐adhesive hemispheres. (b) Synchronization. Finite‐element analysis of wafer stress under eccentric loading shows that a force imbalance of 0.01 N raises the peak von Mises stress by 217%, and the synchronous mechanism achieves a module‐to‐module delay below 10 ms. Independent force‐vs.‐time traces from the four MTP units confirm coordinated triggering. (c) Vacuum‐compatible handling. The gripper picks up, transfers, and places a 45‐µm‐thick silicon wafer inside a 10 Pa low‐pressure chamber (Video S10). (d) Dynamic operational stability. The silicon wafer remains firmly attached without slippage during translation, flipping, and rotation of a six‐axis robotic arm (Video S11). (e) Application summary. The integrated platform achieves a 0.20 s cycle time, damage‐free handling (0 of 10 000 failures; 95% CI, 0 to 0.037%), and vacuum‐compatible operation, which demonstrates that MTP can be deployed as a programmable adhesive‐release interface for the robotic manipulation of ultrathin substrates. Force–time data in (b) are representative of n ≥ 5 independent measurements, and the failure‐rate interval in (e) is a 95% Clopper–Pearson confidence interval.

To test whether MTP scales beyond a single adhesive unit, we assembled a four‐unit gripper on a robotic manipulator for fragile‐substrate handling (Figure 5).

#### 2.6.1 Gripper Design

Using HRPU‐E hemispheres (*R*_a_ = 2.25, radius 2.5 mm), we designed a four‐unit MTP gripper (Figure 5a) for 182 mm × 182 mm silicon wafers of 45 to 150 µm thickness and 4 to 15 g weight. Each unit pairs a central adhesive hemisphere with four surrounding non‐adhesive elastic hemispheres (Figure S31). A total preload of 0.1 N is shared equally among the four adhesive hemispheres, corresponding to 0.025 N per hemisphere. The total triggering threshold of the four‐unit system is 0.348 N (Figure S20), higher than the gravitational load of the handled wafers; this margin lowers the risk of spontaneous release under the wafer's own weight rather than defining a holding capacity, while finite‐element analysis indicates that the peak wafer stress during release remains below the reference fracture‐stress range used in the design analysis. The design builds in redundancy to accommodate varying thickness, compensate for long‐term F_c_ decay of less than 15% after 300 000 cycles, and tolerate inertial disturbances. Optimizing the spacing of the non‐adhesive hemispheres (Figures S32 and S33) reduced peak wafer stress by 30% as the spacing went from 25 to 15 mm; a final spacing of 20 mm was adopted, which gives a 10% stress reduction relative to the initial design (Note S7). The four surrounding non‐adhesive elastic hemispheres serve as mechanical transmission supports for load balancing. The primary driving force for peeling originates from the metastable elastic energy stored at the HRPU‐E hemispherical interface during preloading, while the synchronized triggering module delivers a uniform, minute displacement that initiates detachment.

#### 2.6.2 Synchronous Control

Finite‐element simulation (Note S7) shows that a force imbalance of just 0.01 N among the four units raises the peak von Mises stress by 217% (Figure 5b). We therefore designed a high‐precision synchronous mechanism with a module‐to‐module motion delay below 10 ms. Independent force‐vs.‐time traces confirm unit‐level force responses and near‐synchronous triggering, supporting coordinated operation of the four‐unit gripper and preserving the MTP response under asymmetric loading.

#### 2.6.3 Damage‐Free Wafer Handling

To validate damage‐free handling statistically, we ran 10 000 consecutive cycles of pick‐up, transfer, and release on five independent 45‐µm‐thick wafers (a MTP gripper), and inspected each wafer by PL imaging, SEM, and white‐light interferometry every 1 000 cycles. No microcracks, scratches, or surface damage appeared on any wafer, and the observed failure rate was 0 of 10 000 (95% confidence interval, 0 to 0.037%; Clopper–Pearson method). The gripper also handled wafers of varying thickness from 45 to 150 µm under simulated production conditions (Figures S34, S35 and Videos S7–S9). Pick‐up took 0.07 s, lifting 0.05 s, and release 0.08 s at a trigger speed of 200 mm s^−1^, for a single‐cycle time of about 0.20 s. For context, the measured 0.20 s cycle time approaches the time scale required for high‐throughput single‐wafer handling, although like‐for‐like industrial benchmarking would require standardized testing.

#### 2.6.4 Vacuum and Robotic Integration

Stable pick‐up, transfer, and placement of 45‐µm‐thick wafers was achieved in a 10 Pa low‐vacuum chamber (Figure 5c and Video S10), with an average power consumption below 5 W during operation, which suggests a potential reduction in power use relative to suction‐based handling, subject to system‐level benchmarking. After integration with a six‐axis industrial robotic arm, the gripper held the wafer without slippage through translation, flipping, and rotation (Figure 5d and Video S11), and 100 complete cycles of pick‐up, transfer over 2 s, and release (Videos S12 and S13) left every wafer intact.

Reliable damage‐free handling of ultrathin wafers across thousands of cycles, combined with coordinated pick‐up, programmable release, and robotic manipulation, establishes MTP as a scalable system‐level strategy for the controlled handling of fragile substrates in practical manufacturing environments (Figure 5e).

## 3 Conclusion

This work demonstrates a temporal domain of adhesion control. Whereas existing strategies, from gecko‐inspired microstructures and stimulus‐responsive polymers to metamaterial adhesives with programmable crack paths and snap‐through grippers, modulate interfacial strength directly, metastable transient peeling decouples energy storage from energy release by regulating when stored energy is discharged. The system stores elastic energy during preloading, locks it behind a tunable energy barrier on unloading, and releases it on demand with a minute perturbation. This temporal decoupling mitigates the long‐standing trade‐off between strong bonding and fast release, because the interface maintains holding stability above 20 s yet detaches within 52 ms, two properties that are difficult to achieve together under strength‐domain regulation.

The four‐parameter framework (Δ*E*, *F*_c_, *η*, *H*) provides a quantitative design language for metastable adhesive interfaces. Unlike purely empirical optimization, it links material viscoelasticity, geometry, and operating conditions to measurable performance through scaling relations. The scaling of Δ*E* with modulus—in which softer materials yield deeper metastable wells under identical preload—highlights an underexplored design space for adhesive interfaces, as conventional strength‐domain designs typically prioritize stiffer materials for higher load capacity. The framework is, however, phenomenological. The metastability index requires experimental calibration, and the scaling relations capture observed trends rather than first‐principles predictions, so extension to large‐deformation regimes or complex geometries may require numerical treatment.

The hemispherical geometry plays a role beyond simple contact mechanics. By concentrating stress at the contact perimeter, it promotes edge‐synchronous crack initiation, a geometry‐encoded instability that converts a small perturbation into full‐perimeter simultaneous peeling. This self‐amplifying detachment differs fundamentally from the sequential crack propagation seen in flat or cylindrical geometries. Material viscoelasticity, which provides the energy barrier, and geometric programming, which provides the stress‐concentration mechanism, act together and may extend to other contact geometries and material systems. More generally, we propose that MTP can emerge in systems where three prerequisites are satisfied: a viscoelastic material with sufficient recoverable deformation, a finite interfacial adhesion, and a geometry that localizes edge stress. Within these boundaries, the four‐parameter framework acts as a design rule rather than a description of a single device, framing MTP as a general release principle rather than one optimized gripper.

Several limitations remain. First, MTP has been validated with a single material system (HRPU‐E) on flat, smooth substrates, so extension to rough, curved, or chemically heterogeneous surfaces requires further study. Second, the theoretical framework assumes small deformations near the metastable state and relies on experimentally calibrated parameters. Third, the current gripper handles one wafer at a time, and simultaneous multi‐wafer manipulation would require addressing inter‐unit coupling. Fourth, although the durability over 300 000 cycles is industrially relevant, the mechanism of gradual *F*_c_ degradation of about 15% requires deeper study to enable predictive maintenance. Fifth, the low‐pressure results demonstrate operational compatibility but do not yet decouple the contributions of reduced gas damping, humidity, and surface chemistry.

MTP is not confined to wafer handling. Any task that needs strong temporary bonding followed by rapid, clean release, from transfer printing of flexible electronics to reversible attachment in space robotics, could adopt temporal‐domain adhesion management. The broader point is structural. By separating how strongly an interface holds from when it releases, temporal‐domain regulation adds a design axis orthogonal to existing strength‐domain strategies, one that may be combined with them rather than replacing them. MTP is therefore most appropriately interpreted as a release principle that may be useful wherever strong temporary bonding and rapid clean release must coexist.

## 4 Experimental Section and Methods

### 4.1 Fabrication of HRPU‐E Films and Hemispheres

The fabrication process is illustrated in Figure S3. Briefly, 3.6 mmol of polyether polyol (molecular weight 2000; Yuanye Bio‐Technology, China), 8.1 mmol of IPDI (99%; Macklin, China), 0.1 mmol of tris (2‐aminoethyl) amine (97%; Macklin, China), and 20 µL of dibutyltin dilaurate (95%; Macklin, China) were combined in a flask and reacted at 60°C for 4 h, after which 0.5 wt.% antioxidant (98%; Macklin, China) was added. The precursor was poured into custom molds, degassed under vacuum, cured at 25°C for 40 h, and post‐cured at 60°C for 2 h, yielding HRPU‐E films or hemispheres depending on mold geometry.

### 4.2 Peeling Tests

Adhesion characterization and measurement of the four MTP parameters used a benchtop setup (Figure S36). A force sensor (ZNLBS‐300g, accuracy 0.003 N; Zhongnuo Sensor, China) was mounted on a linear translation stage (TSA300‐B; Zolix, China). For vacuum tests, the linear motor was replaced by a vacuum‐compatible screw motor (KLS60, KECM‐24; Kingsley, China); the vacuum setup is shown in Figure S22. A camera (MV‐CU013; Hikvision, China) recorded and computed the contact area, and an angular displacement stage (GFG9030; Misumi Automation, China) provided angle alignment. Four‐unit synchrony was evaluated on a separate benchtop setup (Figure S37) in which the adhesion–detachment force of each of the four units was recorded and time‐aligned to obtain the per‐unit force–time traces shown in Figure 5b. All tests were performed at 20 ± 1°C and 20 ± 5% RH, with each data point repeated at least five times.

### 4.3 Characterization

Uniaxial tensile and compression tests used a universal testing machine (QT‐6203s; QT Instruments, China). Surface roughness was measured with an optical 3D profilometer (SuperView W1; China National Instruments, China). Microstructure was examined by energy‐dispersive x‐ray spectroscopy and SEM (S4800; Hitachi, Japan). Contact angle and adhesion force were measured with a goniometer (LSA100S‐T; LAUDA Scientific, Germany). A high‐speed camera (SH3‐105; SINCEVISION, China; Figure S38) captured the dynamic peeling. UV tests used a 20 W UV lamp (ZLUV LAMP, China). PL was measured with a PL imaging system (LIS‐R3; BT Imaging, Australia). Dynamic mechanical analysis for the loss factor used a DMA Q800 (TA Instruments, USA). XPS used an ESCALAB 250Xi spectrometer (Thermo Scientific, USA).

### 4.4 Finite‐Element Analysis

The hyperelastic behavior of HRPU‐E was modeled with a third‐order Mooney–Rivlin constitutive law. Uniaxial compression data were fitted by iterative least squares in MATLAB R2024a (MathWorks, USA) to obtain the constitutive parameters (Table S8). For the hemisphere and cylinder simulations, a 0.1 N preload was applied, with the lower contact surface defined as a fixed rigid plane (Note S5). All solid‐mechanics simulations ran in ANSYS 2024 R1 (Workbench; ANSYS, USA).

### 4.5 Pick‐Up and Transfer Tests

The fast pick‐up and transfer manipulator was mounted on a robotic arm (Zu5; JAKA, China) to handle 182 mm × 182 mm silicon wafers of customized thickness (JingAo Solar, China). A high‐definition camera (HD‐4K; ULTRHD, China) recorded all operations, which were performed on a self‐built laboratory‐scale photovoltaic pick‐up line.

### 4.6 Statistical Analysis

All quantitative measurements were repeated at least five times (n ≥ 5) unless otherwise stated, and data are reported as mean ± SD. For the 10 000‐cycle damage‐free validation on five independent wafers, the failure‐rate confidence interval was computed by the Clopper–Pearson exact method at the 95% level. The 95% Clopper–Pearson confidence interval is calculated based on 10 000 operation events and represents the upper bound of the per‐operation failure rate. We note that repeated cycles on the same wafer introduce some correlation, so the reported interval is a conservative upper‐bound estimate of the true failure rate. *F*_c_ fluctuation under environmental conditions is reported as the maximum relative deviation from the mean.

## Author Contributions

L.L. conceived the project. Q.L. and K.Z. conducted the experiments. L.L., Q.L., X.D., X.W., and Y.J. analyzed the data. L.L., Q.L., Y.H., and J.D. prepared the manuscript. L.L., Y.T., and J.D. conceptualized, supervised, provided funding and resource. All authors provided feedback during subsequent revisions.

## Funding

This work was supported by the National Key Research and Development Program of China grant 2023YFB4202600, the Young Scientists Fund of the National Natural Science Foundation of China grant 52405222, the Natural Science Youth Foundation of Jiangsu Province grant BK20230591, and the Postgraduate Research & Practice Innovation Program of Jiangsu Province grant KYCX25_3999.

## Conflicts of Interest

The authors declare no conflicts of interest.

## Supporting information




**Supporting File 1**: advs77766‐sup‐0001‐SuppMat.docx.

**Supporting File 2**: advs77766‐sup‐0002‐VideoS1_1.mp4.

**Supporting File 3**: advs77766‐sup‐0003‐VideoS1_2.mp4.

**Supporting File 4**: advs77766‐sup‐0004‐VideoS2.mp4.

**Supporting File 5**: advs77766‐sup‐0005‐VideoS3.mp4.

**Supporting File 6**: advs77766‐sup‐0006‐VideoS4.mp4.

**Supporting File 7**: advs77766‐sup‐0007‐VideoS5.mp4.

**Supporting File 8**: advs77766‐sup‐0008‐VideoS6.mp4.

**Supporting File 9**: advs77766‐sup‐0009‐VideoS7.mp4.

**Supporting File 10**: advs77766‐sup‐0010‐VideoS8.mp4.

**Supporting File 11**: advs77766‐sup‐0011‐VideoS9.mp4.

**Supporting File 12**: advs77766‐sup‐0012‐VideoS10.mp4.

**Supporting File 13**: advs77766‐sup‐0013‐VideoS11.mp4.

**Supporting File 14**: advs77766‐sup‐0014‐VideoS12.mp4.

**Supporting File 15**: advs77766‐sup‐0015‐VideoS13.mp4.

## Data Availability

The data that supports the findings of this study are available in the supplementary material of this article.
