# Supplementary material for: Bioinspired Metastable Transient Peeling for Repeatable Rapid Release
Source: Adv Sci (Weinh). 2026 Sep 16:e77766. Online ahead of print. doi: 10.1002/advs.77766 (PMC13583243; doi:10.1002/advs.77766)
Supplement: Supplementary file 1 [file ADVS-9999-e77766-s006.docx]

**Supplementary Materials**

**Bioinspired metastable transient peeling for repeatable rapid release**

*Lvzhou Li, Qingyue Li, Yao Hu, Xu Dong, Xiangli Wen, Kai Zhu, Yaoyao Jiang, Yu Tian, Jianning Ding*

E-mail: dingjn@yzu.edu.cn and tianyu@mail.tsinghua.edu.cn

**The file includes:**

Notes S1 to S7.

Figures. S1 to S38.

Tables S1 to S8.

Legends for Videos S1 to S13.

**Other Supplementary Materials for this manuscript include the following:**

Videos S1 to S13

Supplementary Notes

Supplementary Note 1. The derivation process of the energy barrier.

The notations in Table S3 are consistently adopted throughout this Note.

For the contact between a hemisphere and a rigid plane, under conditions of small deformation, the relationship between the characteristic strain rate and the indentation velocity can be approximated as:

 (1)

This approximation applies to small deformation conditions, while for large deformation scenarios, it must be determined through numerical methods.

In this configuration, the substrate is rigid (silicon wafer), and all deformation occurs within the hemispherical indenter. The effective modulus (*E**) is therefore determined by the material of the hemisphere:

$E^{*}=\frac{E}{1-v^{2}}$ (2)

where *E* is the Young’s modulus of the hemispherical material, and *ν* is its Poisson’s ratio.

Hydroxyl functionalization enhances interfacial adhesion with the silicon wafer substrate through the formation of hydrogen bonds at the interface, which manifests as an increase in the interfacial energy *γ*. Within the material, hydroxyl groups can form reversible hydrogen bonds, increasing the viscoelastic dissipation and providing a source of metastability for the system. The derivation in this work treats the existence of the metastable state as a premise, with its effects represented through macroscopic parameters (effective modulus *E*_eff_, relaxation time *τ*, modulus attenuation coefficient *β*_E_, and metastability index *κ*). The total potential energy *Π* of the system consists of three components: the elastic strain energy *U*_e_, the interfacial adhesion energy *U*_a_, and the potential energy of the external force −*Fδ*:

$\Pi\left(\delta,a\right)=U_{e}\left(\delta,a\right)+U_{a}\left(a\right)-F\delta$ (3)

$U_{a}\left(a\right)=-\pi a^{2}\gamma$ (4)

where *δ* is the indentation depth, a is the contact radius, *F* is the external force, *γ* is the interfacial energy (i.e., the work of adhesion per unit area), and *U*_e_ is the elastic strain energy, which depends on the material's constitutive relation and geometry.

In a contact system, *δ* and a are coupled through contact mechanics relationships. By eliminating a, the potential energy described in equation (3) can be expressed as a univariate function of *δ*:

$\Pi\left(\delta\right)=U_{e}\left(\delta\right)+U_{a}\left(\delta\right)-F\delta$ (5)

where in equation (4) *U*_a_ is modified to:

$U_{a}\left(\delta\right)=-\pi a\left(\delta\right)^{2}\gamma$ (6)

The function *a*(*δ*) is determined by contact mechanics.

A metastable state is defined as a local minimum point of the potential energy function, satisfying the conditions:

$\frac{d\Pi}{d\delta}=0,\text{ }\frac{d^{2}\Pi}{d\delta^{2}}<0$ (7)

The energy barrier Δ*E* is defined as the minimum energy required for the system to overcome from the metastable state to the saddle point, which corresponds to the potential energy difference:

$\Delta E=\Pi\left(\delta_{saddld}\right)-\Pi\left(\delta_{meta}\right)$ (8)

where *δ*_meta_ is the indentation depth corresponding to the metastable state, and *δ*_saddle_ is the indentation depth corresponding to the saddle point.

Under quasi-static, dissipation-free conditions, the work done by the external force equals the change in potential energy:

  $Fd\delta=d\Pi$ (9)

Therefore, the energy barrier can also be expressed as:

$\Delta E=\int_{meta}^{\delta_{saddld}} F\left(\delta\right)d\delta$ (10)

To establish an analytical relationship between the energy barrier and the material parameters, the purely elastic, small-deformation limit is first considered. Within this limit, a metastable state still exists (arising from the coupling between interfacial energy and elastic deformation).

For the contact between an elastic hemisphere and a rigid plane, Hertzian theory provides the following relationships:

The relationship between the contact radius and the indentation depth is:

$a^{2}=R\delta$ (11)

Elastic Strain Energy:

$U_{e}=\frac{2}{5}E^{*}R^{1/2}\delta^{5/2}$ (12)

Relationship between external force and indentation depth:

$F=\frac{4}{3}E^{*}R^{1/2}\delta^{3/2}$ (13)

The interfacial adhesion energy is governed by the contact area through geometric relations:

$U_{a}=-\pi a^{2}\gamma=-\pi R\delta\gamma$ (14)

Total potential energy (external force-free state), considering the self-equilibrium state after the removal of the preload (*F*=0), the total potential energy comprises the elastic strain energy and the interfacial adhesion energy:

$\Pi\left(\delta\right)=U_{e}\left(\delta\right)+U_{a}\left(\delta\right)=\frac{2}{5}E^{*}R^{1/2}\delta^{5/2}-\pi R\gamma\delta$ (15)

Metastable position, by differentiating (15) to find the extremum points:

$\frac{d\Pi}{d\delta}=E^{*}R^{1/2}\delta^{3/2}-\pi R\gamma=0$ (16)

The equilibrium position is thus obtained:

$\delta_{eq}=\left(\frac{\pi\gamma}{E^{*}}\right)^{2/3}R^{1/3}$ (17)

Further taking the second derivative of (16):

$\frac{d^{2}\Pi}{d\delta^{2}}=\frac{3}{2}E^{*}R^{1/2}\delta^{1/2}>0\text{ }\left(\delta>0\right)$ (18)

Therefore, *δ*_eq_ corresponds to a minimum of the potential energy, i.e., a metastable state of the system.

When the system detaches from the metastable state to the completely separated state (*δ*=0), the change in potential energy is:

$\Delta E=\Pi\left(0\right)-\Pi\left(\delta_{eq}\right)$ (19)

where *Π*(0)=0 (the potential energy is zero when there is no contact). Substituting (15) and (17) yields:

$\Delta E=\frac{3}{5}\pi^{5/3}\frac{\gamma^{5/3}R^{4/3}}{\left(E^{*}\right)^{2/3}}$ (20)

Thus, the energy barrier at the elastic limit and the material parameters satisfy:

$\Delta E\sim\frac{\gamma^{5/3}R^{4/3}}{\left(E^{*}\right)^{2/3}}$ (21)

For viscoelastic materials, the analytical solution under the elastic limit is no longer strictly valid, due to the following reasons: the modulus varies with the loading rate; the strain energy *U*_e_ depends not only on the current deformation but also on the deformation history; and viscoelastic dissipation may lead to multiple metastable states.

Nevertheless, the scaling relationship for the energy barrier can be established through the following analysis:

The physical parameters affecting the energy barrier include the interfacial energy *γ*, the effective modulus *E*_eff_, and the characteristic geometric length scale *R*. From dimensional analysis, the general form of the energy barrier can be expressed as:

 (22)

where *C* is a dimensionless constant, and *α* is an undetermined exponent.

Under the elastic limit, Equation (21) yields a scaling exponent$\alpha=-2/3$, with a proportionality constant $C=\frac{3}{5}\pi^{5/3}$. The value of this exponent is determined by the following physical mechanisms: Energy balance: the detachment process must simultaneously overcome the interfacial energy and the elastic strain energy; Fracture mechanics analogy: the critical energy release rate is *G_c_*=*γ*, and the elastic energy release rate scales as $G\sim F^{2}/\left(ER^{2}\right)$; Geometric constraint: the relationship between the contact radius and the indentation depth follows $a\sim\sqrt{R\delta}$.These mechanisms collectively lead to a power-law relationship with an exponent $\alpha=-2/3$.

For viscoelastic materials, the modulus should be understood as the effective modulus, which depends on the loading rate and satisfies: In the quasi‑static limit (), *E*_eff_ → *E*_0_ (the static modulus); In the high‑rate limit (), *E*_eff_→*E*_∞_ (the glassy modulus); increases monotonically with the loading rate. Combining the above analysis, the scaling relationship between the energy barrier and the material parameters for a viscoelastic hemispherical contact system is:

 (23)

In the purely elastic, small-deformation limit, the proportionality constant is denoted as $\frac{3}{5}\pi^{5/3}$.For the viscoelastic system investigated in this work, this constant may deviate due to large deformations and rate-dependent effects, and must therefore be determined through experimental calibration.

Supplementary Note 2. Derivation of the critical trigger force

The following derivation is based on the existence of a metastable state within the system. The critical triggering force, *F*_c_, is defined as the minimum external force required to drive the system from the metastable state over the energy barrier, thereby triggering interfacial instability.

As detailed in Supplementary Note 1, the energy barrier Δ*E* is defined as the potential energy difference between the metastable state and the saddle point (Eq.10). Let the characteristic displacement Δ*δ* be defined as:

$\Delta\delta=\delta_{saddle}-\delta_{meta}$ (24)

Then the following scaling relation holds:

 (25)

From Eq. (23) in Supplementary Note 1, the characteristic displacement Δ*δ* has the dimension of length. Based on dimensional analysis, its general form is:

$\Delta\delta\sim\gamma^{-c}\left(E_{eff}\right)^{c}R^{1+c}$ (26)

where *c* is an indeterminate exponent.

Substituting (23) and (26) into (25) yields:

$F_{c}\sim\frac{\Delta E}{\Delta\delta}\sim\gamma^{5/3+c}R^{1/3-c}\left(E_{eff}\right)^{-2/3-c}$ (27)

In systems exhibiting a metastable state, the exponent *c* is constrained by physical conditions: The characteristic displacement decreases with increasing modulus, i.e., Δ*δ*∼(*E*_eff_)*^c^*, implying *c* < 0. The critical triggering force is positively correlated with the interfacial energy, requiring the exponent for *γ*, which is (5/3 + *c*), to be greater than 0, i.e., *c* > -5/3. Furthermore, the critical triggering force is positively correlated with the effective modulus (in the presence of metastability, a stiffer material results in a higher energy barrier for the metastable state, leading to a larger triggering force). This requires the exponent for *E*_eff_, which is (-2/3 - *c*), to be greater than 0, i.e., c < -2/3. Consequently:

$-\frac{5}{3}<c<-\frac{2}{3}$ (28)

Let $c=-\frac{2}{3}-\kappa$, where *κ* represents the metastability index. Substituting this into Eq. (27) yields:

$F_{c}\sim\gamma^{1-\kappa}R^{1+\kappa}\left(E_{eff}\right)^{\kappa}$ (29)

where 0 < *κ* < 1 (derived from the condition c > -5/3, which leads to *κ* < 1).

Finally, the critical triggering force *F*_c_ is obtained as:

$F_{c}\propto\gamma^{1-\kappa}R^{1+\kappa}\left(E_{eff}\right)^{\kappa}$ (30)

According to Eq. (30), the scaling relationship between the critical triggering force *F*_c_ and the material parameters is summarized as follows:

Supplementary Note 3. Derivation of the holding force decay rate

The holding force decay rate *η* is defined as the rate at which the holding force decreases over time under a constant displacement (or constant external force) condition due to viscoelastic relaxation. Thus, *η* is expressed as:

 (31)

Holding force decay occurs under conditions where the system remains in contact and detachment has not yet taken place. This process does not involve the crossing of a metastable energy barrier; rather, it reflects the intrinsic viscoelastic relaxation behavior of the material. For the hydroxyl-functionalized rigid crosslinked polyurethane elastomer, the relaxation mechanisms include molecular chain segment motion, the rupture and reformation of reversible physical crosslinks, and the disentanglement of the entanglement network.

According to Hertzian contact theory, for the contact between an elastic hemisphere and a rigid plane.

$F=\frac{4}{3}E^{*}R^{1/2}\delta^{3/2}$ (32)

Under a constant displacement condition *δ*_0_:

$F\left(t\right)=\frac{4}{3}E^{*}\left(t\right)R^{1/2}\delta_{0}^{3/2}$ (33)

Therefore,

$\frac{F\left(t\right)}{F\left(0\right)}=\frac{E^{*}\left(t\right)}{E^{*}\left(0\right)}$ (34)

For the crosslinked rigid polyurethane elastomer, the standard linear solid model is employed to describe its relaxation behavior:

$E^{*}\left(t\right)=E_{\infty}+\left(E_{0}-E_{\infty}\right)e^{-t/\tau}$ (35)

Here, *E*_0_= *E**(0) is the instantaneous modulus; *E*_∞_ is the equilibrium modulus (long-time limit); and *τ* is the characteristic relaxation time.

The time-dependent function for force decay is:

$\frac{F(t)}{F_{0}}=\frac{E_{\infty}+\left(E_{0}-E_{\infty}\right)e^{-t/\tau}}{E_{0}}$ (36)

This function describes the process of holding force decay from its initial value *F*_0_ to the equilibrium value *F*_∞_.

The initial decay rate is defined as:

 (37)

Define the modulus attenuation coefficient as:

$\beta_{E}=\frac{E_{0}-E_{\infty}}{E_{0}}$ (38)

Therefore,

$\eta=\frac{\beta_{E}}{\tau}$ (39)

Supplementary Note 4. Derivation of the hysteresis coefficient

The hysteresis coefficient *H* is defined as the ratio of the energy dissipated to the total input energy during a loading-unloading cycle. In a system exhibiting a metastable state, the unloading process may involve detachment by overcoming the metastable energy barrier; consequently, the dissipation associated with this barrier contributes to the hysteresis coefficient.

Definition of the Hysteresis Coefficient:

$H=\frac{W_{diss}}{W_{input}}$ (40)

Total dissipation comprises both pure viscoelastic dissipation and metastable energy barrier dissipation.

$W_{diss}=W_{diss}^{(visco)}+W_{diss}^{(meta)}$ (41)

According to Supplementary Note 2 and Equation (30) for the critical triggering force, the input work is:

 (42)

Under the standard linear solid model, the proportion of pure viscoelastic dissipation to the total input work is:

 (43)

where$\beta_{E}=\left(E_{0}-E_{\infty}\right)/E_{0}$ is the modulus attenuation coefficient, reflecting the proportion of the material's relaxable component;is the Deborah number, representing the ratio of the loading rate to the material's relaxation time; and the term $D_{e}/1\text{+}D_{e}$ is a rate-dependent factor, which increases with the loading rate and eventually saturates. This expression gives the pure viscoelastic hysteresis coefficient, i.e., the ratio of pure viscoelastic dissipation to the total input work. When *D_e_*→0 (quasi-static), *H*_visco_→0, indicating vanishing viscoelastic dissipation. When *D_e_*→∞ (high speed), *H*_visco_=*β_E_*/2, meaning the viscoelastic dissipation reaches its maximum. At *D_e_*=1, *H*_visco_=*β_E_*/2, where the dissipation is half of its maximum value.

The dissipation due to the metastable energy barrier is equal to the energy barrier Δ*E*:

$W_{diss}^{meta}\sim\Delta\Pi\sim\frac{\gamma^{5/3}R^{4/3}}{\left[E_{eff}\right]^{2/3}}$ (44)

Therefore, the ratio of metastable barrier dissipation to the total input work is:

$H_{meta}=\frac{W_{diss}^{meta}}{W_{input}}\sim\frac{\gamma^{5/3}R^{4/3}/\left[E_{eff}\right]^{2/3}}{\gamma^{1-\kappa}}=\frac{\gamma^{2/3+\kappa}R^{1/3-\kappa}}{\left[E_{eff}\right]^{2/3+\kappa}\delta_{\max}}$ (45)

The total hysteresis coefficient is the sum of the pure viscoelastic hysteresis coefficient and the metastable hysteresis coefficient:

 (46)

where the first term is the pure viscoelastic hysteresis coefficient, representing the intrinsic viscoelastic dissipation of the material; the second term is the additional hysteresis contributed by the metastable energy barrier; *κ*_₂_ is a constant to be determined, which requires experimental calibration; and *κ* is the metastability index defined in Supplementary Note 2, with 0 < *κ* < 1.

Supplementary Note 5. FEA Simulation of HRPU-E Cylinder and Hemisphere

A finite element model of the HRPU-E cylinder and hemisphere was established using ANSYS/Workbench 2024 for solid mechanics simulation. The model primarily consists of HRPU-E and a structural steel substrate, simulating the behavior of the HRPU-E cylinder and hemisphere under preload applied on a smooth surface. The structural steel substrate has dimensions of 10 mm in length, 2 mm in thickness, and 10 mm in width. The HRPU-E cylinder has a diameter of 5 mm and a height of 2.75 mm. The HRPU-E hemisphere has a radius of 2.5 mm and a height of 2.75 mm. In this simulation, the bottom surface of the structural steel substrate is fully fixed. The structural steel substrate and the HRPU-E cylinder/hemisphere are initially not in contact. The HRPU-E cylinder and hemisphere are set with dynamic meshing, and a force-controlled load is applied to the top surface of the adhesive material. The hyperelastic mechanical behavior of HRPU-E is defined using a third-order Mooney-Rivlin constitutive model, with parameters provided in Table S3. By varying the magnitude of the preload, the differences in stress distribution between the HRPU-E cylinder and hemisphere under preload are investigated.

Supplementary Note 5. Calculation Procedure for the Four Parameter

Testing the critical triggering force. As shown in Figure. 2a, a preload force (*F*_preload_) is applied to the viscoelastic hemisphere to establish intimate contact. Subsequently, the preload is withdrawn, and a holding force is applied and maintained for a duration (*T*_hold_). Peeling is then triggered at a specified velocity (*V*_peeling_), and the measured peak force is taken as the critical triggering force. Corresponding tests are performed by varying the parameters *F*_preload_, *T*_hold_, and *V*_peel_.

Calculation of the energy barrier. As illustrated in Figure. 2a, the energy barrier is approximated based on Eq. (10) in Supplementary Note 1. This is achieved by integrating the force *F* and the peeling velocity *V*_peeling_ over the detachment instability interval, that is, integrating the product F V_peeling_ over time (equivalently the work integral). The calculated energy barrier is given by:

$\Delta E=\int_{t_{1}}^{t_{2}} F(t)V_{peel}(t)dt=\int_{\delta_{1}}^{\delta_{2}} F(\delta)d\delta$ (47)

Calculation of the holding force decay rate. Based on an approximation of the energy barrier derived from Eq. (36) in Supplementary Note 3, the holding force decay rate can be obtained as:

 (48)

where *F*_c_(0) refers to the critical triggering force at the moment the holding time is zero, and *F*_c_(*t*) refers to the critical triggering force at holding time *t*. In practice, *η* was estimated from the decay of the measured critical triggering force after different holding times, which serves as an operational proxy for the decay of metastable holding stability.

Calculation of the hysteresis coefficient *H*. As illustrated in Figure. 2a, the hysteresis coefficient is approximated based on Eq. (45) in Supplementary Note 4, yielding:

$H=\frac{W_{loading}-W_{unloading}}{W_{loading}}$ (49)

Supplementary Note 7. FEA Simulation of the BMTP Gripper

A solid mechanics model of the MTP gripper acting on a silicon wafer was established using the finite element software ANSYS/Workbench 2024. The silicon wafer dimensions are 182 mm × 182 mm with a thickness of 90 μm. The HRPU-E hemispherical adhesive region experiences a peeling force, with a total normal peeling force of 0.64 N (a conservative load exceeding the experimentally measured four-unit triggering threshold of 0.348 N) applied to the silicon wafer, while the non-adhesive hemispherical regions were set as fixed constraints. By varying the number of HRPU-E hemispheres and non-adhesive hemispheres, the influence of the number of adhesion–detachment units on the stress applied to the silicon wafer was investigated. By changing the distance between non-adhesive hemispheres, the effect of non-adhesive hemisphere spacing on silicon wafer stress was explored. By altering the magnitude of the peeling force between HRPU-E hemispheres, the impact of eccentric loading on silicon wafer stress was studied. The 90-μm wafer was selected as a representative intermediate thickness for stress-distribution analysis. The most fragile 45-μm wafers were validated experimentally through 10,000 damage-free pick-up, transfer, and release cycles without observed damage; a dedicated 45-μm finite-element model would provide a more conservative stress estimate.

**Supplementary Figures:**

**Figure S1. Characterization of silicon wafer roughness.**

**Figure S2.** **Synthetic route for the HRPU-E elastomer.**

**Figure S3. Fabrication process of HRPU-E material.**

**Figure S4. Fourier transform infrared spectroscopy analysis.**

**Figure S5. XPS characterization of the HRPU-E surface.**

**Figure S6. Comparison of finite element Von-Mises stress for hemispherical and cylindrical adhesive structures.**

**Figure S7. Peel force curves of conventional detachment and MTP.**

**Figure S8. Evolution of contact area during peeling for conventional detachment and MTP.**

**Figure S9. Triggering force (*F*_c_) as a function of trigger velocity for conventional detachment and MTP.**

**Figure S10. Uniaxial tensile curves of HRPU-E with different molar ratios of IPDI to polyether polyol.**

**Figure S11. JKR contact mechanics test setup.**

**Figure S12. Loss factor of HRPU-E.**

**Figure S13. Filamentation during de-adhesion.**

**Figure S14.** **Effect of *R*_a_ on the holding time.**

**Figure S15. Force – time curves of quasi-static tensile tests.**

**Figure S16. Displacement – time curves of quasi-static tensile tests.**

**Figure S17. Potential energy–displacement curves reconstructed via numerical integration.**

**Figure S18. Effect of preload on *F*_hold_.**

**Figure S19. Evolution of contact states under different preload forces.**

**Figure S20. Effect of preload on *F*_c_.**

**Figure S21. Effect of surface energy on *F*_c_.**

**Figure S22. XPS C1s spectra of the adhesive hemisphere before and after 100 cycles**

**Figure S23. 3D white light interference comparison of contact points on crystalline silicon wafers before and after gripper pick-up. (A)** 3D white light interference topography of contact points before gripper pick-up. **(B)** 3D white light interference topography of contact points after gripper pick-up and placement.

**Figure S24. SEM comparison of contact points on wafer before and after gripper pick-up. (A)** SEM micrograph of contact points on the wafer before pick-up by the gripper. **(B)** SEM micrograph of contact points on the wafer after pick-up and placement by the gripper.

**Figure S25. PL comparison of crystalline silicon wafers contacted by adhesive gripper vs. bernoulli chuck.** (A) PL image of crystalline silicon wafer before pick-up by adhesive manipulator. (B) PL image of crystalline silicon wafer after pick-up and placement by adhesive gripper.

**Figure S26. Adhesion performance data of HRPU-E from time-dependent aging tests.**

**Figure S27. Comparison of O1s XPS spectra of HRPU-E before and after 1400 min UV irradiation.**

**Figure S28. Water contact angle.**

**Figure S29. Comparison of O1s XPS spectra of HRPU-E before and after water rinsing.**

**Figure S30. Physical image of the self-designed vacuum environment setup.**

**Figure S31. Boundary stress control structure. (A)** Top view of the boundary stress control structure composition. **(B)** Side view of the boundary stress control structure composition.

**Figure S32. Optimization of spacing between the four non-adhesive elastic hemispheres.**

**Figure S33. Stress distribution comparison at varied spacings.** (**A**) Von-Mises stress at 25-mm spacing. (**B**) Shear stress at 25-mm spacing. (**C**) Von-Mises stress at 20-mm spacing; (**D**) Shear stress at 20-mm spacing; (**E**) Von-Mises stress at 15-mm spacing; (**F**) Shear stress at 15-mm

**Figure S34. Pickup of silicon wafers with different thicknesses by a gripper.**

**Figure S35. The pick-up step took 0.07 s, the lifting took 0.05 s, and the release took 0.08 s at a triggering speed of 200 mm/s.**

**Figure S36. Experimental setup for adhesive performance testing.**

**Figure S37.Test setup for gripper synchrony characterization**

**Figure S38. High-speed camera captures of detachment dynamics.**

**Supplementary Tables:**

**Table S1. Comparison of representative reversible adhesive systems across regulatory mechanisms and performance metrics.**

| **Category** | **Representative mechanism** | **Regulatory dimension** | **Energy input timing** | **Typical release time** | **Core working principle** |
| --- | --- | --- | --- | --- | --- |
| Viscoelastic peeling ^[18,37,38]^ | Rate-dependent detachment | Strength domain | Continuous during peeling | 200–1000 ms | Modulation of interfacial resistance via rate-dependent modulus |
| Microstructure switching ^[2,10,14,17,20,40]^ | Gecko-inspired fibrillar adhesion | Strength domain | Continuous during detachment | 200–500 ms | Contact area modulation via microstructural deformation |
| Stimulus-responsive adhesion ^[9,15,23,27,39,41]^ | Thermo-/magneto-responsive modulus | Strength domain | Continuous stimulus during release | 1000–10,000 ms | Interfacial strength tuning via modulus change under external fields |
| *This work* | Metastable transient peeling | Temporal domain | Pre-stored before triggering | 52 ms | Timing control of pre-stored interfacial energy via a tunable energy barrier |

**Table S2 Comparative Analysis of Performance between the MTP Gripper and Existing Adhesive Grippers**

| **Ref.** | **Release time/ms** | **Number of times** | **Vacuum compatibility** |
| --- | --- | --- | --- |
| [2] | 267 | 10000 | Not investigated |
| [20] | 210 | 30 | Not investigated |
| [27] | 500 | 250 | Incompatible |
| [41] | 240 | 50 | Incompatible |
| [42] | 300 | 200 | Not investigated |
| [23] | 500 | 50 | Not investigated |
| [39] | 200 | 50 | Incompatible |
| [17] | 250 | 100 | Incompatible |
| [18] | 850 | 50 | Incompatible |
| [40] | 900 | 50 | Incompatible |
| This work | 52 | 300000 | Compatible |

**Table S3 Correspondence table of notations**

| Symbol | Meaning | Unit |
| --- | --- | --- |
| *γ* | Interface energy | J/m² |
| *R* | Hemispherical radius | m |
| *E* | Young's modulus | Pa |
| *ν* | Poisson's ratio | dimensionless |
| *E*^∗^ | Plane strain modulus | Pa |
|  | Rate-dependent effective modulus | Pa |
|  | Characteristic strain rate | s⁻¹ |
|  | Indentation velocity | m/s |
| *τ* | Characteristic relaxation time | s |
| *De* | Deborah number | dimensionless |
| *E*_0_ | Instantaneous modulus | Pa |
| *E*_∞_ | Equilibrium modulus | Pa |
| *β_E_* | Modulus attenuation coefficient | dimensionless |
| *β* | Critical trigger force enhancement coefficient | dimensionless |
| *δ* | Indentation depth | m |
| *δ*_max_ | Maximum indentation depth | m |
| *a* | Contact radius | m |
| *F* | External force | N |
| *F_c_* | Critical trigger force | N |
| Δ*E* | Energy barrier | J |
| *η* | Holding force decay rate | s⁻¹ |
| *H* | Hysteresis coefficient | dimensionless |
| *κ* | Metastable index，0<κ<1 | dimensionless |

**Table S4 Summary of trend relationships between the energy barrier and various parameters revealed by Eq. (23).**

| Parameter | Change Direction | Effect on Δ*E* | Physical Explanation |
| --- | --- | --- | --- |
| *γ* | ↑ | ↑ | Stronger adhesion requires more energy for detachment. |
| *R* | ↑ | ↑ | A larger contact area confines more elastic potential energy. |
| *E*_eff_ | ↑ | ↓ | A stiffer material deforms less under the same load, storing less energy. |
|  | ↑ | ↓ | The barrier is indirectly reduced through an increase in *E*_eff_. |

**Table S5 Scaling relationships between the critical triggering force *F*_c_ and material parameters derived from Eq. (30)**

| Parameter | Change Direction | Effect on *F*_c_ | Physical Explanation |
| --- | --- | --- | --- |
| *γ* | ↑ | ↑ | Stronger adhesion requires a larger triggering force. |
| *R* | ↑ | ↑ | A larger contact area enhances adhesive force. |
| *E*_eff_ | ↑ | ↑ | F_c_ increases indirectly through the increase of *E*_eff_. |
|  | ↑ | ↑ | Material hardening increases the metastable energy barrier. |
| *κ* | ↑ | ↑ | A more pronounced metastable effect leads to a larger triggering force. |

**Table S6 Scaling relationships between the holding force decay rate *η* and material parameters derived from Eq. (39)**

| Parameter | Change Direction | Effect on *F*_c_ | Physical Explanation |
| --- | --- | --- | --- |
| *τ* | ↑ | ↓ | Slower relaxation leads to a slower force decay rate. |
| *E*_0_ | ↑ | ↑ | A higher modulus provides a greater reservoir of stress available for relaxation. |
| *E*_∞_ | ↑ | ↓ | A higher residual modulus corresponds to a smaller extent of relaxation. |
| *E*_0_−*E*_∞_ | ↑ | ↑ | A larger relaxable modulus component results in a faster decay rate. |

**Table S7 Summary of scaling relationships between the hysteresis coefficient *H* and material parameters based on Eq. 46**

| Symbol | Effect on *H*_visco_ | Effect on *H*_meta_ | Overall Trend |
| --- | --- | --- | --- |
| *β_E_* | ↑ | No effect | ↑ |
| *τ* | ↑ | No effect | ↑ |
|  | ↑ | ↓ | Competitive Effects |
| *γ* | No effect | ↑ | ↑ |
| *R* | No effect | ↑ | ↑ |
| *E*eff | No effect | ↓ | ↓ |
| *δ*_max_ | No effect | ↓ | ↓ |
| *κ* | No effect | Complex | Requires experimental determination |

**Table S8 Four MTP parameters of viscoelastic HRPU-E hemispheres at different Ra values (n ≥ 5)**

| *R*_a_ | *E*_∞_ (MPa) | *E*_a_ (kPa) | Δ*E* (μJ) （*t*=20 s） | *F*_c_ (N)  （*t*=20 s） | *η* (%/s)（*t*=20 s） | *H* | tanδ (25 °C) | *T*_hold_*(s)* |
| --- | --- | --- | --- | --- | --- | --- | --- | --- |
| 2.00 | 0.04 | 167.71 | 1212.66 | 2.13 | 0.32 | 0.44 | 0.38 | 140 |
| 2.16 | 0.13 | 134.78 | 145.16 | 0.75 | 1.26 | 0.67 | 0.25 | 60 |
| 2.25 | 0.32 | 107.47 | 6.61 | 0.10 | 3.50 | 0.73 | 0.18 | 20 |
| 2.33 | 0.42 | 39.51 | 0.78 | 0.05 | 100 | 0.82 | 0.13 | 3 |
| 2.66 | 0.73 | 1.51 | 0.002 | 0.01 | 100 | 1 | 0.08 | 0 |

**Table S9. Fitted Parameters of Mooney-Rivlin Third-Order Constitutive Model for Uniaxial Compression Tests**

| **IPDI/polyether** | ***C_10_*(MPa)** | ***C_01_*(MPa)** | ***C_20_*(MPa)** | ***C_11_*(MPa)** | ***C_02_*(MPa)** |
| --- | --- | --- | --- | --- | --- |
| 2.25 | 0.0697 | -0.0598 | 32.6214 | -52.8246 | 22.3098 |

**Supplementary Videos:**

Supplementary Video 1

High-speed camera to capture changes in detachment

Supplementary Video 2

The contact states of cylindrical and hemispherical structures are compared

Supplementary Video 3

Detachment process of cylindrical and hemispherical adhesive structures

Supplementary Video 4

Intermittently recorded at every 20,000 cycles during 300,000 adhesion–detachment tests

Supplementary Video 5

High-speed camera captures silicon wafer detachment process

Supplementary Video 6

Surface adhesion test

Supplementary Video 7

Pickup of silicon wafers of varying thicknesses

Supplementary Video 8

Application in a photovoltaic pick-and-place pilot line

Supplementary Video 9

Fast continuous pick-and-place of silicon wafers

Supplementary Video 10

Transfer silicon wafers in a vacuum environment

Supplementary Video 11

Multi-attitude manipulation of grasped silicon wafers

Supplementary Video 12

100 cycles of adhesion–detachment transfer of a silicon wafer

Supplementary Video 13

10,000 cycles of adhesion–detachment of silicon wafers
